# Online vestibular rehabilitation for chronic vestibular syndrome: 36-month follow-up of a randomised controlled trial in general practice

**DOI:** 10.3399/BJGP.2022.0468

**Published:** 2023-07-25

**Authors:** Vincent A van Vugt, Hà TN Ngo, Johannes C van der Wouden, Jos WR Twisk, Henriëtte E van der Horst, Otto R Maarsingh

**Affiliations:** Department of General Practice, Amsterdam UMC location Vrije Universiteit Amsterdam; Amsterdam Public Health Research Institute, Amsterdam.; Department of General Practice, Amsterdam UMC location Vrije Universiteit Amsterdam; Amsterdam Public Health Research Institute, Amsterdam.; Department of General Practice, Amsterdam UMC location Vrije Universiteit Amsterdam; Amsterdam Public Health Research Institute, Amsterdam.; Department of Epidemiology and Biostatistics, Amsterdam UMC location Vrije Universiteit Amsterdam; Amsterdam Public Health Research Institute, Amsterdam.; Department of General Practice, Amsterdam UMC location Vrije Universiteit Amsterdam; Amsterdam Public Health Research Institute, Amsterdam.; Department of General Practice, Amsterdam UMC location Vrije Universiteit Amsterdam; Amsterdam Public Health Research Institute, Amsterdam.

**Keywords:** dizziness, follow-up studies, physiotherapy, primary care, vertigo, vestibular diseases

## Abstract

**Background:**

Vestibular rehabilitation (VR) is the preferred treatment for chronic vestibular symptoms such as dizziness and vertigo. An internet-based programme was developed to increase uptake of VR. The authors have previously reported that internet-based VR resulted in a clinically relevant decrease of vestibular symptoms for up to 6 months, compared with usual care.

**Aim:**

To evaluate long-term outcomes of internet-based VR in patients with chronic vestibular syndrome.

**Design and setting:**

A randomised controlled trial was conducted in Dutch general practice involving 322 participants aged ≥50 years with chronic vestibular syndrome. Participants were randomised to stand-alone VR, blended VR (with physiotherapy support), and usual care. Usual care participants were allowed to cross over to stand-alone VR 6 months after randomisation.

**Method:**

Participants were approached 36 months after randomisation. The primary outcome was the presence of vestibular symptoms as measured by the vertigo symptom scale—short form (VSS–SF). Secondary outcomes were dizziness-related impairment, anxiety, depressive symptoms, and healthcare utilisation.

**Results:**

At 36-month follow-up, 65% of participants filled in the VSS–SF. In the usual care group, 38% of participants had crossed over to VR at 6 months. There were no significant differences in vestibular symptoms between VR groups and usual care (mean difference = −0.8 points, 95% confidence interval [CI] = −2.8 to 1.2, for stand-alone VR; −0.3, 95% CI = −2.2 to 1.7, for blended VR). In VR groups, clinically relevant improvement compared with baseline was maintained over time.

**Conclusion:**

Internet-based VR provides a maintained improvement of vestibular symptoms for up to 36 months in patients with chronic vestibular syndrome.

## INTRODUCTION

GPs often encounter patients with vestibular symptoms, such as dizziness and vertigo.^1^ In the general population the lifetime prevalence of vestibular symptoms varies between 17% and 30%.^2^^,^^3^ Older patients generally experience more frequent and severe symptoms than younger patients and have an increased chance of developing chronic symptoms (known as chronic vestibular syndrome in the current nomenclature).^2^^,^^4^^,^^5^ The preferred treatment for chronic vestibular syndrome is vestibular rehabilitation (VR), but this exercise-based treatment is still underused.^6^^–^^8^ VR consists of specific exercises designed to maximise central nervous system compensation after vestibular disorders occur. In addition, a form of exposure-based behaviour therapy takes place by provoking vestibular symptoms in a controlled context.^9^^,^^10^ To increase uptake of VR, an internet-based intervention was developed. In 2017, this online treatment was first shown to be effective in a randomised controlled trial in the UK by researchers at the University of Southampton.^10^^,^^11^ The authors of the present article worked together with researchers at the University of Southampton to create a Dutch version of internet-based VR and subsequently conducted a large, randomised controlled trial in the Netherlands investigating the effectiveness of internet-based VR both without (stand-alone VR) and with support (blended VR).^12^ The authors previously reported that, compared with usual care, internet-based VR decreased vestibular symptoms for up to 6 months in patients with chronic vestibular syndrome in Dutch primary care.^13^ In this article, the authors report the 36-month outcomes.

## METHOD

### Study design

A pragmatic, three-armed, individually randomised controlled trial was conducted involving 59 Dutch general practices. The Consolidated Standards of Reporting Trials (CONSORT) reporting guidelines for non-drug treatment interventions were followed and the trial was registered in the Netherlands Trial Register (reference: NTR5712). A detailed research protocol and the results of the short-term effectiveness analysis at 6 months were published previously.^12^^,^^13^ Participants were recruited between June 2017 and July 2018. For this pre-specified long-term 36-month follow-up, participants were approached again 3 years after randomisation between August 2020 and July 2021.

### Participants

Electronic medical records were used to identify patients aged ≥50 years who visited their GP with a vestibular symptom in the past 2 years. The GP screened potentially eligible participants to exclude those with medical contraindications for making the required head movements, serious comorbid conditions that precluded participation in an exercise programme, an identifiable non-vestibular cause of their symptoms, or current enrolment in a related study. Trial information and a form to express interest was provided to potential participants. Physicians in the research team checked the eligibility criteria of interested patients by telephone. The inclusion criteria were:

- good command of the Dutch language;
- access to the internet and an email account;
- persisting vestibular symptoms at time of inclusion, present for ≥1 month; and
- vestibular symptoms exacerbated or triggered by head movements.

**Table:** How this fits in

Vestibular rehabilitation (VR) is an exercise-based therapy that can be used to decrease vestibular symptoms, such as dizziness and vertigo, by gradually stimulating the vestibular system. Although VR is the preferred treatment for chronic vestibular syndromes, the intervention is still largely underused in clinical practice. In a randomised controlled trial in general practice, internet-based VR was previously shown to result in a clinically relevant decrease of vestibular symptoms over 6 months. In this 36-month follow-up study, the authors found no significant differences in vestibular symptoms between internet-based VR groups and usual care, but clinically relevant effects after VR were maintained over time.

These inclusion criteria were used to identify participants with a chronic vestibular syndrome, as defined by the Bárány Society in the International Classification of Vestibular Disorders,^5^^,^^14^ who were deemed suitable to receive online VR (Supplementary Table S1).

### Randomisation and blinding

After the informed consent procedure, participants were sent an email with a link to the trial website. On completion of the baseline questionnaire, the software program allocated them to internet-based VR (stand-alone VR), internet-based VR with support (blended VR), or usual care. The randomisation process was fully automated and concealed from the research team. Due to the nature of the trial interventions, it was not possible to blind participants, physiotherapists, and research assistants.

### Interventions

#### Stand-alone internet-based VR

Stand-alone VR participants received access to the Vertigo Training internet-based intervention. Vertigo Training was a Dutch translation of the safe and effective British internet-based VR intervention Balance Retraining (freely available from https://balance.lifeguidehealth.org).^11^ Vertigo Training lasted 6 weeks and consisted of weekly online sessions and daily VR exercises, explained in more detail in the previously published short-term effectiveness analysis^13^ and Appendix (Supplementary Table S2). Stand-alone VR participants received the standard level of care from their own GP without any restrictions.

#### Blended internet-based VR with physiotherapy support

Blended VR participants were granted access to the same online Vertigo Training intervention as the stand-alone VR participants. In addition, they were visited twice at home by a trained physiotherapist during the 6-week intervention period. These 45-minute physiotherapy sessions took place in the first and third week. The physiotherapist provided information about the background of vestibular symptoms and VR, and talked about doubts and concerns the participant might have. They also taught participants how to use the online intervention, took participants through a set of VR exercises, advised on coping with obstacles to adherence, and encouraged participants to continue the exercises. Blended VR participants also received the standard level of care from their own GP with no restrictions.

#### Usual care

Usual care participants received standard care from their own doctor without restrictions. The authors provided participating doctors with written instructions, asking them to diagnose and treat all causes of vestibular symptoms according to the guidelines of the Dutch College of General Practitioners.^15^ After completing the short-term follow-up measures at 6 months, all usual care participants were offered access to stand-alone VR. Usual care participants who chose to cross over at this time were registered in a separate database.

### Primary and secondary outcomes

Measurements were collected at baseline, 3-, 6-, and 36-months’ follow-up. At baseline, participants provided information on their age, sex, level of education, living situation, comorbidities, vestibular diagnosis, frequency and average duration of vestibular symptoms, and the time since their vestibular disorder was diagnosed. The primary outcome was vestibular symptoms as measured by the vertigo symptom scale—short form (VSS–SF).^16^^,^^17^ The questionnaire can be used to measure the frequency and severity of 15 vestibular symptoms, with more points indicating more frequent/severe symptoms (range 0–60). The VSS–SF has been used in several large VR trials to measure treatment effect (clinically relevant difference ≥3 points).^11^^,^^18^^–^^20^ The questionnaire has high internal consistency (Cronbach’s α = 0.90), excellent discriminative ability (area under the curve = 0.87), and high test–retest reliability (intraclass correlation coefficient = 0.88).^17^ The secondary outcomes were the Dizziness Handicap Inventory questionnaire (DHI) for dizziness-related impairment;^21^ and the Patient Health Questionnaire (PHQ)^22^ for severity of anxiety (Generalised Anxiety Disorder Assessment [GAD-7] subscale)^23^ and depressive symptoms (PHQ-9 subscale).^24^ For the 36-month follow-up, all primary and secondary outcome measures were assessed again, except for the single dichotomous item of subjective improvement compared with baseline. In addition, enquiries were made about the participants’ healthcare use and long-term adherence to the exercises. Participants were asked if they had visited any healthcare professional for their vestibular symptoms since the last follow-up measure. The stand-alone VR and blended VR participants were also asked how long they continued using the VR exercises after receiving access. Usual care participants who chose to cross over to stand-alone VR at 6 months were registered in a separate database.

### Statistical analysis

An intention-to-treat analysis was performed to compare stand-alone VR and blended VR versus usual care. A linear mixed-model analysis was used to account for repeated measures within individuals. In a longitudinal dataset, this technique accounts for missing data without performing multiple imputations.^25^ This analysis included group assignment, time, and the interaction between group assignment and time. By creating dummy variables, time and group assignment were included as categorical variables, which enabled the calculation of outcomes for each follow-up moment while adjusting for baseline values.^26^ The same potential confounders were adjusted for as in the 6-month analysis.^13^ Because part of the usual care group started stand-alone VR at 6 months, an additional post-hoc analysis was conducted to assess outcomes of cross-over participants separately from other usual care participants at different time points. The same variables were used for this analysis as for the main analysis. Several post-hoc sensitivity analyses were also conducted to assess non-response bias and attrition. For non-response, differences in characteristics were descriptively analysed between participants who filled in the 36-month follow-up measurement and those who did not. A complete case analysis was also performed by repeating the primary and secondary outcome analysis without participants who did not fill in the 36-month follow-up measure. Lastly, to further assess the effects of attrition, a per-protocol analysis was completed with participants who filled in the 36-month follow-up. This included only the participants assigned to stand-alone VR who completed all six online sessions, and the participants assigned to blended VR who completed all six online sessions and both physiotherapist visits. Stata (version 14.1) was used for statistical analyses.

## RESULTS

### Primary outcome

**Figure 1.**
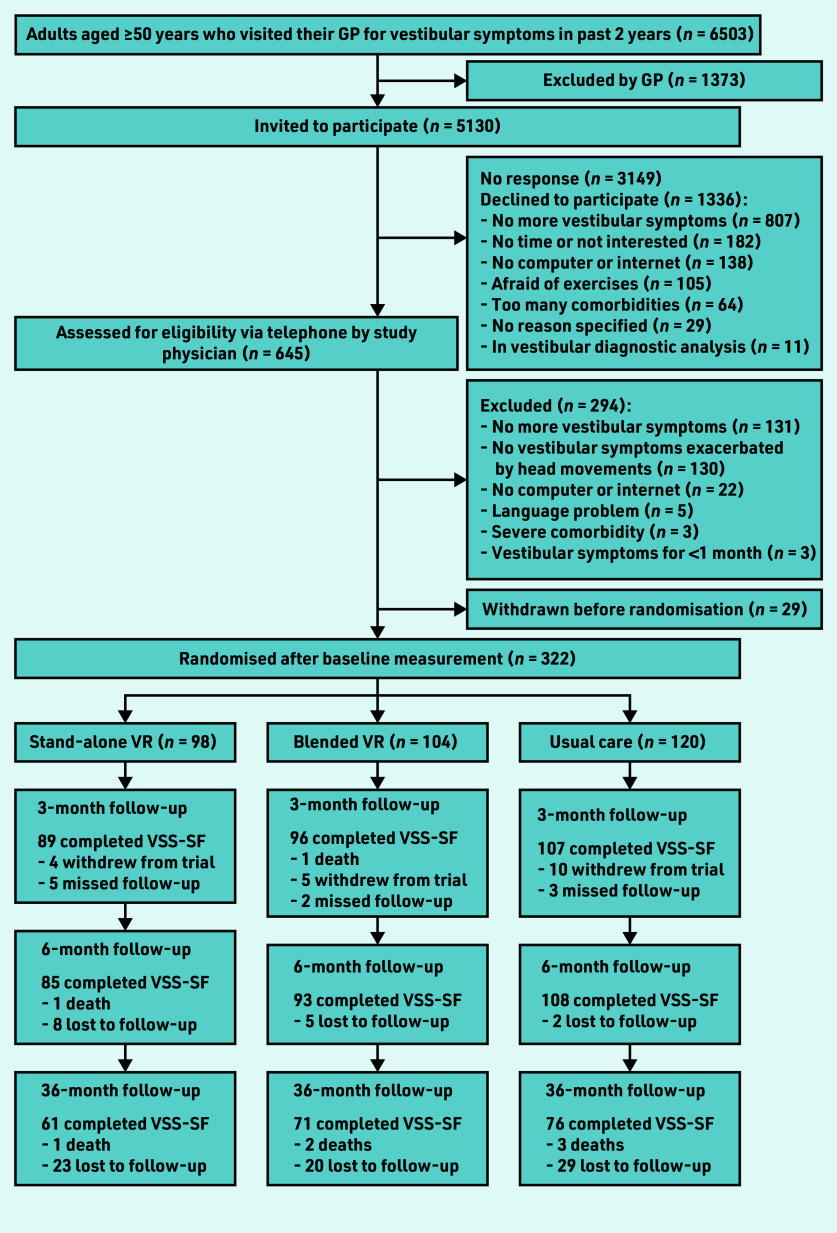
*Flow of participants through trial. VR = vestibular rehabilitation. VSS–SF = vertigo symptom scale–short form.*

**Figure 2.**
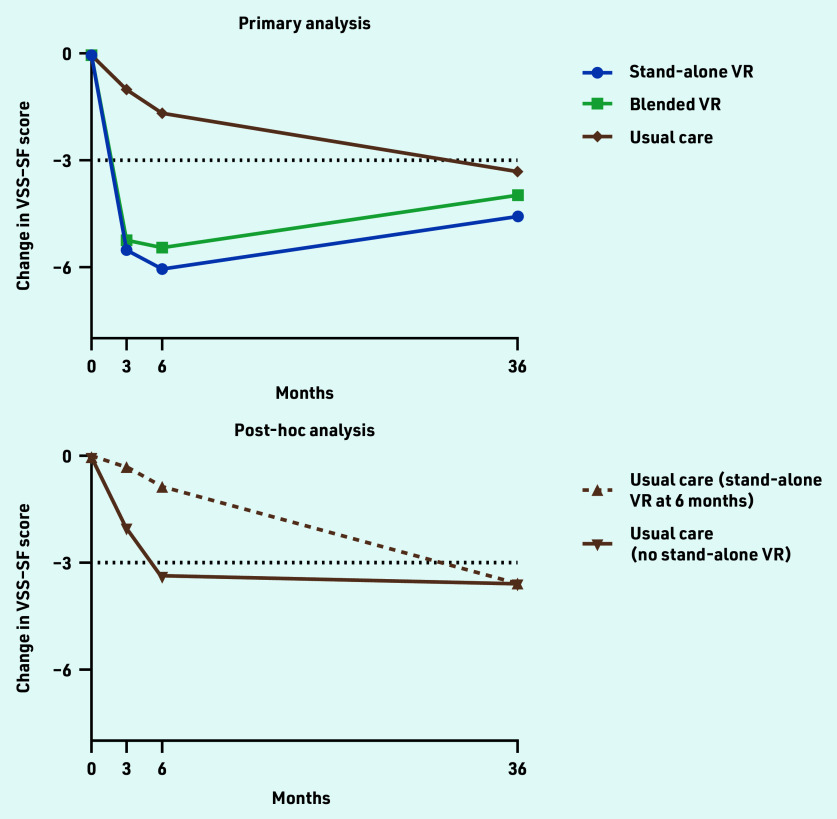
*Primary outcome 36-month follow-up results after VR. VSS–SF range is 0-60, higher values indicate more severe/frequent vestibular symptoms, clinically relevant difference ≥3 points (indicated by dotted black line). Primary analysis: all participants analysed according to allocation (total,* N *= 322; stand-alone VR,* n *= 98; blended VR,* n *= 104; and usual care,* n *= 120). Post-hoc analysis: usual care at 3-year follow-up (*n *= 76) and comparison of participants who did not cross over to stand-alone VR after 6 months (*n *= 47) versus those who did (*n *= 29). All analyses were adjusted for baseline VSS–SF values, repeated measurements within participants’ age, gender, level of education, living situation, number of chronic diseases, time since vestibular diagnosis, and the presence of a panic disorder, generalised anxiety disorder, or major depressive disorder at baseline.*

At baseline, 322 participants were randomised (baseline characteristics described in Table 1). At 36-month follow-up, 208 participants (65%) filled in the VSS–SF (Figure 1). Follow-up rates were similar across treatment groups (stand-alone VR, *n* = 61/98, 62%; blended VR, *n* = 71/104, 68%; and usual care, *n* = 76/120, 63%). In this long-term follow-up, there was no significant differences in vestibular symptoms between the VR groups and the usual care group (Table 2). Improvements in vestibular symptoms were largely maintained in stand-alone VR (−4.6 points, 95% CI = −6.0 to −3.1) and blended VR (−4.0 points, 95% CI = −5.5 to −2.4) participants. However, usual care participants also showed clinically significant improvement compared with baseline (−3.3 points, 95% CI = −4.8 to −1.8). The post-hoc analysis showed that usual care participants who never used VR (62%) improved spontaneously over the first 6 months (−3.4 points, 95% CI = −5.3 to −1.4) and showed no further improvement at 3 years (−3.6 points, 95% CI = −5.5 to −1.6). In contrast, usual care participants who crossed over (38%) had hardly improved in the first 6 months (before they started stand-alone VR) (−0.8 points, 95% CI = −3.5 to 1.9), but showed significant improvement at 3 years (−3.6 points, 95% CI = −6.3 to −0.8). Figure 2 shows the changes in VSS–SF score during the trial.

**Table 1.** Baseline characteristics of participants^13^

**Characteristic**	**Stand-alone VR (*n* = 98), *n* (%)^a^**	**Blended VR (*n* = 104), *n* (%)^a^**	**Usual care (*n* = 120), *n* (%)^a^**	**Total sample (*N* = 322), *n* (%)^a^**
**Age, years, mean (SD)**	66.7 (9.5)	67.4 (9.8)	67.0 (9.4)	67.0 (9.5)

**Sex, female**	64 (65)	69 (66)	64 (53)	197 (61)

**Level of education**				
Low	33 (34)	37 (36)	36 (30)	106 (33)
Middle	25 (26)	31 (30)	30 (25)	86 (27)
High	40 (41)	36 (35)	54 (45)	130 (40)

**Living situation**				
Alone	34 (35)	33 (32)	35 (29)	102 (32)
With partner	64 (65)	71 (68)	85 (71)	220 (68)

**Number of chronic diseases^b^**				
0	59 (60)	64 (62)	63 (53)	186 (58)
1	28 (29)	32 (31)	41 (34)	101 (31)
2	8 (8)	4 (4)	12 (10)	24 (7)
≥3	3 (3)	4 (4)	4 (3)	11 (3)

**Time since vestibular diagnosis^c^**				
1–6 months	15 (15)	22 (21)	13 (11)	50 (16)
6 months to 2 years	28 (29)	27 (26)	39 (33)	94 (29)
2–10 years	31 (32)	44 (42)	48 (40)	123 (38)
>10 years	23 (24)	11 (11)	18 (15)	52 (16)

**Self-reported vestibular diagnosis^c^**				
No known diagnosis	67 (68)	69 (66)	77 (64)	213 (66)
BPPV	11 (11)	17 (16)	22 (18)	50 (16)
Ménière’s disease	9 (9)	9 (9)	10 (8)	28 (9)
Vestibular neuritis	6 (6)	4 (4)	7 (6)	17 (5)
PPPD	0 (0)	1 (1)	0 (0)	1 (0.3)
Other^d^	4 (4)	4 (4)	2 (2)	10 (3)

**Disorders at baseline according to PHQ^e^**	14 (14)	16 (15)	23 (19)	53 (16)
Panic disorder	2 (2)	3 (3)	5 (4)	10 (3)
Generalised anxiety disorder	12 (12)	15 (14)	19 (16)	46 (14)
Major depressive disorder	5 (5)	6 (6)	9 (8)	20 (6)

a *Unless otherwise stated.*

b *Chronic non-specific lung disease, cardiac disease, peripheral arterial disease, stroke, diabetes mellitus, arthritis, and cancer.*

c *Data on variable were missing for 3 participants: stand-alone VR (*n *= 1) and usual care (*n *= 2).*

d *Other diagnoses reported by patients consisted of traumatic brain injury, cerebrovascular accident, Parkinson’s disease, bacterial meningitis, and vestibular organ surgical procedure.*

e *Some patients had multiple psychiatric disorders. BPPV = benign paroxysmal positional vertigo. PHQ = Patient Health Questionnaire. PPPD = persistent postural-perceptual dizziness. SD = standard deviation. VR = vestibular rehabilitation.*

**Table 2.** Comparison primary outcome between treatment groups at 36 months

**Primary analysis^c^**	**Change from baseline, mean (95% CI)^a^**			**Comparison, adjusted mean difference (95% CI)^a^^,^^b^**	

	**Stand-alone VR**	**Blended VR**	**Usual care**	**Stand-alone VR versus usual care**	**Blended VR versus usual care**

VSS-SF baseline, mean (SD)	14.1 (8.9)	13.8 (8.3)	13.2 (8.6)	—	—
3 months	−5.5 (−6.8 to −4.2)	−5.2 (−6.6 to −3.8)	−1.0 (−2.3 to 0.3)	−4.3 (−5.9 to −2.6)	−3.9 (−5.5 to −2.3)
6 months	−6.0 (−7.3 to −4.7)	−5.5 (−6.9 to −4.0)	−1.6 (−3.0 to −0.3)	−4.1 (−5.8 to −2.5)	−3.5 (−5.1 to −1.9)
36 months	−4.6 (−6.0 to −3.1)	−4.0 (−5.5 to −2.4)	−3.3 (−4.8 to −1.8)	−0.8 (−2.8 to 1.2)	−0.3 (−2.2 to 1.7)

**Post-hoc analysis^d^**	**Usual care (no stand-alone VR at 6 months)**	**Usual care (stand-alone VR at 6 months)**			

VSS-SF baseline, mean (SD)	11.2 (7.3)	13.4 (8.7)			
3 months	−2.0 (−4.0 to −0.1)	−0.3 (−3.0 to 2.4)			
6 months	−3.4 (−5.3 to −1.4)	−0.8 (−3.5 to 1.9)			
36 months	−3.6 (−5.5 to −1.6)	−3.6 (−6.3 to −0.8)			

a *Unless otherwise stated.*

b *Adjusted for baseline values, repeated measurements within participants’ age, gender, level of education, living situation, number of chronic diseases, time since vestibular diagnosis, and the presence of a panic disorder, generalised anxiety disorder, or major depressive disorder at baseline.*

c *Primary analysis: all participants analysed according to allocation (total,* N *= 322; stand-alone VR,* n *= 98; blended VR,* n *= 104; and usual care,* n *= 120).*

d *Post-hoc analysis: usual care at 3-year follow-up (*n *= 76) and comparison of participants who did not cross over to stand-alone VR after 6 months (*n *= 47) versus those who did (*n *= 29). SD = standard deviation. VR = vestibular rehabilitation. VSS–SF = vertigo symptom scale–short form.*

### Secondary outcomes

There were no significant differences in impairment from dizziness between VR groups and usual care at 36 months (Table 3). However, similar to vestibular symptoms, improvement of dizziness-related impairment was maintained at 36 months in the stand-alone VR (−9.9 points, 95% CI = −13.3 to −6.4) and blended VR groups (−10.4, 95% CI = −13.3 to −7.5). In the post-hoc analysis, usual care participants who crossed over to VR at 6 months showed an improvement of dizziness-related impairment thereafter (6 months: −6.6 points, 95% CI = −12.8 to −0.4; 36 months: −11.0, 95% CI = −17.3 to −4.7), whereas participants who did not cross over spontaneously improved at 6 months and maintained that improvement at 36 months (6 months: −10.1 points, 95% CI = −14.5 to −5.7; 36 months: −10.8, 95% CI = −15.2 to −6.4). There were no significant differences in anxiety and depressive symptoms between VR groups and usual care after 36 months. Significant improvement of anxiety symptoms was maintained over time in both VR groups, while improvement of depressive symptoms was only maintained in the blended VR group. In the usual care group significant improvement of depressive symptoms was seen at 36 months, but not at previous time points.

**Table 3.** Comparison of secondary outcomes between treatment groups at 36 months

**Primary analysis secondary outcomes^c^**	**Change from baseline, mean (95% CI)^a^**			**Comparison, adjusted mean difference (95% CI)^a^^,^^b^**	

	**Stand-alone VR versus usual care**	**Blended VR versus usual care**	**Usual care**	**Stand-alone VR versus usual care**	**Blended VR versus usual care**

**DHI baseline, mean (SD)**	34.8 (18.5)	36.0 (21.9)	35.8 (19.9)	–	–
3 months	−9.9 (−13.0 to 6.9)	−9.5 (−12.2 to −6.9)	−5.4 (−8.2 to −2.6)	−4.6 (−8.3 to −0.9)	−3.8 (−7.5 to −0.2)
6 months	−11.5 (−14.6 to −8.5)	−11.6 (−14.2 to −8.9)	−6.9 (−9.6 to −4.1)	−4.6 (−8.3 to −0.9)	−4.4 (−8.1 to −0.7)
36 months	−9.9 (−13.3 to −6.4)	−10.4 (−13.3 to −7.5)	−10.2 (−13.3 to −7.0)	0.4 (−3.9 to 4.7)	−0.0 (−4.2 to 4.1)

**GAD-7 baseline, mean (SD)**	3.7 (4.6)	4.0 (4.3)	4.5 (4.8)	–	–
3 months	−0.5 (−1.2 to 0.1)	−1.0 (−1.5 to −0.4)	0.3 (−0.4 to 1.0)	−1.1 (−1.9 to −0.3)	−1.4 (−2.2 to −0.6)
6 months	−1.0 (−1.7 to −0.3)	−1.2 (−1.8 to −0.6)	−0.2 (−0.9 to 0.6)	−1.1 (−1.9 to −0.3)	−1.2 (−2.0 to −0.4)
36 months	−0.8 (−1.6 to −0.1)	−1.0 (−1.6 to −0.4)	−1.3 (−2.1 to −0.5)	0.2 (−0.7 to 1.2)	0.2 (−0.7 to 1.1)

**PHQ-9 baseline, mean (SD)**	4.5 (4.7)	5.4 (4.5)	5.9 (5.4)	–	–
3 months	−0.2 (−1.0 to 0.6)	−1.0 (−1.6 to −0.3)	−0.2 (−1.0 to 0.7)	−0.6 (−1.5 to 0.4)	−0.9 (−1.8 to 0.1)
6 months	−0.8 (−1.6 to 0.0)	−1.4 (−2.1 to −0.8)	−0.7 (−1.6 to 0.1)	−0.6 (−1.6 to 0.4)	−0.8 (−1.7 to 0.2)
36 months	0.0 (−0.9 to 1.0)	−1.1 (−1.8 to −0.3)	−1.6 (−2.6 to −0.6)	1.2 (0.0 to 2.3)	0.4 (−0.7 to 1.5)

**Post-hoc analysis secondary outcomes^d^**	**Usual care (no stand-alone VR at 6 months)**	**Usual care (stand-alone VR at 6 months)**			

**DHI baseline, mean (SD)**	29.3 (18.4)	37.5 (21.0)			
3 months	−5.9 (−10.3 to −1.4)	−7.2 (−13.4 to −1.1)			
6 months	−10.1 (−14.5 to −5.7)	−6.6 (−12.8 to −0.4)			
36 months	−10.8 (−15.2 to −6.4)	−11.0 (−17.3 to −4.7)			

**GAD-7 baseline, mean (SD)**	4.0 (4.8)	5.0 (5.3)			
3 months	0.7 (−0.5 to 1.9)	0.4 (−1.0 to 1.8)			
6 months	−1.1 (−2.4 to 0.1)	0.3 (−1.0 to 1.7)			
36 months	−1.7 (−2.9 to 0.5)	−0.9 (−2.3 to 0.4)			

**PHQ-9 baseline, mean (SD)**	5.1 (4.8)	6.2 (5.9)			
3 months	−0.1 (−1.5 to 1.2)	−0.3 (−1.9 to 1.3)			
6 months	−1.4 (−2.8 to 0.0)	−0.6 (−2.1 to 1.0)			
36 months	−1.4 (−2.8 to 0.0)	−2.2 (−3.8 to −0.6)			

a *Unless otherwise stated.*

b *Adjusted for baseline values, repeated measurements within participants age, gender, level of education, living situation, number of chronic diseases, time since vestibular diagnosis, and the presence of a panic disorder, generalised anxiety disorder, or major depressive disorder at baseline.*

c *Primary analysis: all participants analysed according to allocation (total,* N *= 322; stand-alone VR,* n *= 98; blended VR,* n *= 104; and usual care,* n *= 120).*

d *Post-hoc analysis: usual care at 3-year follow-up (*n *= 76) and comparison of participants who did not cross over to stand-alone VR after 6 months (*n *= 47) versus those who did (*n *= 29). DHI = dizziness handicap inventory. GAD-7 = generalised anxiety disorder assessment-7. PHQ-9 = patient health questionnaire-9. SD = standard deviation. VR = vestibular rehabilitation.*

Additionally, participants were asked about their healthcare use in the period between the 6-month and 36-month follow-up (Table 4). No relevant differences were detected between VR groups and usual care in visits to the GP, GP out-of-hours service, medical specialist, emergency department, and hospital admissions for vestibular symptoms. A few patients were admitted to the hospital (stand-alone VR, *n* = 4 and usual care, *n* = 1). Reasons for admission were ‘transient ischaemic attack and epileptic attack’ (*n* = 1), ‘fall’ (*n* = 1), ‘atrial fibrillation’ (*n* = 1), and ‘not reported’ (*n* = 2). No patient mentioned adverse events related to VR exercises.

**Table 4.** Visits to healthcare providers at 36-month follow-up and reported adherence to VR exercises

**Healthcare use between 6- and 36-months’ follow-up**	**Stand-alone VR (*n* = 61), *n* (%)**	**Blended VR (*n* = 71), *n* (%)**	**Usual care (*n* = 76), *n*(%)**	**Total sample (*N* = 208), *n* (%)**
GP	18 (30)	14 (20)	17 (22)	49 (24)
GP out-of-hours service	11 (18)	13 (18)	14 (18)	38 (18)
Medical specialist	14 (23)	19 (27)	28 (37)	61 (29)
Emergency department	4 (7)	1 (1)	2 (3)	7 (3)
Hospital admission	4 (7)	0 (0)	1 (1)	5 (2)
**Adherence to VR exercises in VR treatment groups**				
**Time participants regularly used VR exercises after trial start**	**Stand-alone VR (*n* = 61), *n* (%)**		**Blended VR (*n* = 71), *n* (%)**	**Stand-alone + blended VR (*n* = 132), *n* (%)**
1–6 weeks	21 (34)		27 (38)	48 (36)
6 weeks to 3 months	13 (21)		11 (15)	24 (18)
3–6 months	7 (11)		5 (7)	12 (9)
6–12 months	4 (7)		11 (15)	15 (11)
>12 months	10 (16)		11 (15)	21 (16)
Not filled in	6 (10)		6 (8)	12 (9)
**Reported use of VR exercises after 3 years**				
Never	24 (39)		25 (35)	49 (37)
A few times	23 (38)		26 (37)	49 (37)
Several times	8 (13)		15 (21)	23 (17)
Quite often	3 (5)		3 (4)	6 (5)
Very often	3 (5)		2 (3)	5 (4)

*VR = vestibular rehabilitation.*

Lastly, participants were asked to elaborate on their adherence to VR after the trial (Table 4). Even though the treatment lasted for 6 weeks, 18% of participants continued to use VR regularly up to 3 months after start of the trial and 16% of the participants used VR regularly for >12 months. At 36 months, 63% of the participants who completed the questionnaire still continued to perform VR exercises.

## Post-hoc sensitivity analyses for non-response bias and attrition

To assess non-response bias and attrition the authors conducted several sensitivity analyses. The baseline characteristics for participants who filled in the 36-month follow-up and those who did not are described separately in Supplementary Table S3. Participants who completed 36-month follow-up had a relatively higher level of education, were less likely to live alone, and more often followed the per-protocol treatment. The results of a subsequent complete case sensitivity analysis, where only the patients who filled in the 36-month were included, showed similar results to the primary analysis (Supplementary Table S4). Lastly, a per-protocol analysis with participants who filled in the 36-month follow-up showed that VR participants who followed all sessions had fewer vestibular symptoms at long-term follow-up, indicating a positive effect of better engagement (Supplementary Table S5).

## DISCUSSION

### Summary

At 36-month follow-up, vestibular symptoms did not significantly differ between participants who received internet-based VR and usual care. The same applies with regard to impairment experienced due to dizziness, anxiety, and depressive symptoms. Nevertheless, clinically relevant improvements in internet-based VR groups were maintained over time. Recovery of usual care participants who crossed over at 6 months further indicates the value of online VR when vestibular symptoms do not improve spontaneously. There were no clear differences in healthcare utilisation between groups at long-term follow-up, and serious adverse events due to VR were unlikely. Many patients reported continuing VR exercises long after the end of the 6-week intervention period.

### Strengths and limitations

This study has several strengths. This was a large, well-designed trial and participants showed good adherence to the intervention.^13^ Furthermore, the primary outcome, the VSS–SF, is a validated patient-reported outcome measure that is used in most major VR trials.^17^^,^^27^ Another strength is the high response rate in this long-term follow-up in a sample of older patients that can be prone to attrition.

There are also some limitations. The response rate at 6-month follow-up was higher than at 36-month follow-up. The number of participants at 36-month follow-up was lower than the 80 participants per group in the original power calculation for 6-month follow-up.^13^ Post-hoc power analyses are generally not considered useful.^28^ Because this is the first long-term follow-up of VR, to the authors' knowledge, it is also impossible to estimate the expected treatment effect. However, the lower long-term follow-up rate may have made it harder to detect significant differences. The results presented in this long-term follow-up may not be representative for all, as the post-hoc sensitivity analyses showed that participants who were lost to follow-up were relatively lower educated and more likely to live alone. It is therefore important to pay special attention to these groups. The per-protocol analysis showed that following all online VR sessions may positively influence results, so finding ways to improve engagement for all will be vital to successful implementation. Another limitation for the comparison between groups was the cross over of participants in the usual care group. The decision to allow participants to cross over was made before the trial to increase the benefits of inclusion for participants, but it complicated the comparison between VR groups and usual care in this long-term follow-up. Lastly, this study did not collect data on specific treatments for dizziness received by participants during the follow-up period. Nevertheless, given the relative absence of effective treatments available in chronic vestibular syndrome, it is unlikely that this strongly influenced results.

### Comparison with existing literature

To the authors’ knowledge, this is the first randomised controlled trial of VR with a long-term follow-up.^29^^,^^30^ Outcomes of VR for vestibular disorders have been evaluated extensively, but trials have mainly covered a follow-up period of up to 1 year.^29^^–^^32^ Internet-based VR is a relatively new concept, but it has been investigated before in a randomised controlled trial in the UK.^11^ In the UK trial, internet-based VR was compared with usual care in adults aged ≥50 years. Results from the UK trial were similar to this study: VSS–SF at 6 months significantly improved compared with baseline (2.26 points difference, 95% CI = 0.39 to 4.12). However, the follow-up period in the UK study was 6 months, whereas the follow-up period in this study was 36 months. More recently, a web application has been developed in Norway for patients with persistent postural-perceptual dizziness, which is also classified as a chronic vestibular syndrome.^33^ This small and uncontrolled trial also showed clinically relevant improvement of vestibular symptoms after 3 months (6.9 points difference in VSS–SF score at 3 months, 95% CI = 0.2 to 13.6). Long-term outcomes of this web application have not been assessed yet.

### Implications for research and practice

Results from this trial show that internet-based VR can provide a long-term reduction of vestibular symptoms in primary care patients with chronic vestibular syndrome. The favourable conclusions of this 36-month follow-up show that this safe and inexpensive treatment is ready to be made available for daily general practice. Future research questions are therefore mostly aimed at implementation. The authors are currently working on a large implementation research project to make stand-alone VR freely available to all in Dutch general practice (I-RECOVER trial). By assessing adoption, coverage, and sustainability of online VR, the authors aim to bridge science and daily general practice, providing patients and GPs with an easy solution to treat chronic vestibular syndrome.

## Data

Data are available on reasonable request. Requests for sharing study data can be made on specific grounds, either 1) with the aim of corroborating the study results in the interest of public health, or 2) in the context of an audit by a competent authority. Sufficient information needs to be provided to confirm that the request is made for one of the above-mentioned purposes, including a sound justification and, in case of a request with a view to corroborate study results, a protocol on the research for which the data will be used or a plan for quality control checks, as applicable.
